# Generation Z usage of Instagram for TV: investigating media consumption, user interaction patterns, and mental health outcomes

**DOI:** 10.3389/fpubh.2026.1919452

**Published:** 2026-09-17

**Authors:** Mohammed Habes, Khadija Farhan Alhumaid, Ali Al Hadeed, Razaz Waheeb Attar, Kevin Ayoubi

**Affiliations:** 1 Faculty of Mass Communication, Radio & TV Department, Yarmouk University, Irbid, Jordan; 2 Rabdan Academy, Abu Dhabi, United Arab Emirates; 3 Public Relations and Advertising Department, Faculty of Mass Communication, Yarmouk University, Irbid, Jordan; 4 Management Department, College of Business Administration, Princess Nourah Bint Abdulrahman University, Riyadh, Saudi Arabia; 5 Head of General Education Department, Sharjah Maritime Academy, Khor Fakkan, United Arab Emirates

**Keywords:** building trust, digital impact on mental health, digital wellbeing, Generation Z, Instagram reels, knowledge sharing

## Abstract

**Introduction:**

The fast development of social media platforms has changed the way Generation Z communicates, sharing information and consuming digital material. One of such innovations is Instagram Reels, a popular short and long video-sharing functionality that helps with communication, knowledge exchange, and content usage. The feature was first introduced as Instagram TV (IGTV) in 2018 and subsequently integrated into Instagram Reels during the 2022 product consolidation of Instagram, however, the user behaviors underlying this topic, including video content consumption, creator engagement, and trust building, are still the center of the existing video ecosystem on the platform. Although the use of video-based content on Instagram is becoming increasingly popular, scarce studies have considered which factors affect its use and the consequences of this usage on the mental health and psychological wellbeing of users.

**Objective:**

The paper examines the predictors of Instagram Reels use and the connection between Instagram Reels and mental health outcomes in Generation Z users. Based on Social Influence Theory, Expectation Confirmation Theory and the Technology Acceptance Model (TAM), a conceptual framework was created with consideration of Social Influence, Expectation Confirmation, Behavioral Intention, Idea Sharing, Building trust, Instagram Reels Usage, and Mental Health Outcomes.

**Methods:**

The quantitative research design was used and a survey with 422 Generation Z users in Jordan was carried out using a structured questionnaire. Partial Least Squares Structural Equation Modeling (PLS-SEM) and Artificial Neural Network (ANN) were the methods that were used to analyze the data.

**Results:**

The results showed that, Social Influence and Expectation Confirmation play significant roles in Idea Sharing and Building Trust which consequently leads to the increased use of Instagram Reels. Behavioral Intention was cited and found to predict the relationship between Idea Sharing, Trust Building and platform engagement. Moreover, the Usage of Instagram Reels was also revealed to have a substantial impact on Mental Health Outcomes, with both positive and negative wellbeing indicators demonstrating varying effects. The Importance-Performance Map Analysis (IPMA) has determined Idea Sharing to be the most significant driver of Instagram Reels Usage, then Social Influence and Expectation Confirmation.

**Conclusion:**

The paper finds that interactive features and perceived benefits of social media platform have a pivotal role in determining users engagement and psychological experiences. The results add to the body of literature on social media adoption and digital wellbeing and offer some practical implications to educators, policymakers, and platform developers interested in creating healthier digital spaces in which Generation Z users can operate.

## Introduction

Digital technology has been greatly integrated into modern communication systems, changing how people receive information, socialize, and receive media content (1). The use of internet-based technologies in the operation of traditional media platforms like television, radio, and newspapers has led to a vastly connected digital media space (2). This intersection has increased the pace at which social networking sites (SNSs) are evolving beyond being considered communication platforms but as a revolutionary enhancement of the existing media systems (3). In line with the idea of McLuhan that the medium is the message, digital platforms have become the continuation of the already existing communication technologies and at the same time, transform the social interactions and cultural practices (4). The fast growth of social media sites has drastically transformed daily life such that digital communication has become an inseparable part of social, educational, and entertainment (5, 6). In the eyes of the Mediatization Theory, digital media have become so ingrained into social institutions and everyday life that they have a fundamental impact on how people make meaning, form relationships and identities (84). Andreas Hepp argues that human experience gets mediated by digital technologies more and more, changing patterns of communication and even social reality; this conceptual framework of mediate views the approach to the analysis of Instagram Reels as an institutionally located, and not exclusively technical, communication space (7). Social media like Facebook, Instagram, X (previously Twitter), YouTube, and similar content sharing networks have enabled the most opportunities of interaction, self-expression, and information exchange & Impact on Behavioral Intention and Mental Health Outcomes (8). Video-sharing technologies have seen phenomenal growth among such platforms. YouTube, Vimeo and DailyMotion are providing opportunities to users to create, share and consume audiovisual content to learn, be informed and entertained (9). In more modern times, convergence in technology has helped to create a combination of social networking features with video services that resemble TV (10). A case in point was the introduction of Instagram TV (IGTV) in 2018 that changed Instagram, a platform that primarily served to share photos and short films, into a long-form video-sharing platform (11). In 2022, Instagram officially phased out IGTV as a separate feature and integrated its long- and short-form video capabilities into Instagram Reels, which has become the main content creation, discovery, and consumption platform in the platform (10). This current feature—Instagram Reels—is explored in the given study by relying on the conceptual foundations of the previous research in the IGTV era as the underlying mechanisms of interest (video-based content consumption, interaction with creators, and trust in the community) remain the same in this product transition (12).

Both Fadillah and Long (13) and Hadi and Destiwati (14) argue that the integration of long- and short-form video capabilities in Instagram Reels contributes to mental health problems among Generation Z, which have seen a significant increase globally and regionally (15, 106). Studies indicate rising rates of anxiety, depression, loneliness, and fear of missing out (FOMO), particularly among heavy social media users. The literature also reveals gender-related differences, with females tending to report higher levels of anxiety and depression symptoms. Furthermore, the impact of use varies depending on the patterns of interaction with digital platforms. Comparisons between social media users and non-users show that the intensity and quality of interaction, rather than mere use, are associated with mental health outcomes (83). This trend is also evident in the Middle East, where the widespread adoption of digital platforms among young people has led to increased interest in studying their psychological and social effects, underscoring the importance of examining the factors linking Instagram Reels use to the mental health of Generation Z (2, 105, 108).

The long-form and algorithmically curated video content provided the users with more opportunities to create content, consume media, and engage with each other and made the platform more appealing to younger users (16). The rise of the popularity of Instagram Reels has been noticeable in Generation Z, a generation that has been exposed to digital technologies at a young age. The past studies have also noted that using Instagram has a number of advantages, such as improved communication, social connectivity, learning, and user-generated content (12). However, the growing adoption of digital media into the lives of adolescents and young adults has raised a lot of concerns on its possible psychological impacts (107). Recent research is gradually implying that an excessive use of screen-based technologies can influence the emotional wellbeing, cognitive growth, self-esteem, and mental health outcomes (17, 107).

These issues are in line with the results of the article Screens and the Developing Mind: Digital Technology, Mental Health, and Wellbeing in Children and Adolescents that claims that the effects of digital technologies on the psychological development of young people are complex and multidimensional (107). Screen-based media experiences instead of creating consistently positive or negative effects, they interact with processes of development, social, and individual influence in the formation of mental health and wellbeing (18). Overexposure to the content of social media has been linked to increased anxiety, depressive symptoms, social comparison, fear of missing out (FOMO), broken sleep habits, and decreased psychological wellbeing in adolescents and young adults. Simultaneously, social support, experimenting with identity, self-expression, and community interaction can be offered through digital platforms when they are used in considered and meaningful amounts (19).

These issues are especially topical in the context of Instagram Reels as the service is a hybrid of the immersive nature of viewing in the style of traditional television with the interactive nature of social media and the idea of social comparison (20). In contrast to traditional TV, Instagram Reels promotes active interaction in terms of comments, likes, sharing content and algorithmically organized information, which may aggravate the emotional and cognitive experience of users (1). To Generation Z, in which the everyday experiences of the individual are highly connected to the consumption of digital media, the long-term exposure to the content of Instagram Reels can shape the values of self-esteem, the quality of social interactions, emotional health, and mental health in general (99, 100, 109).

Although there is an increasing literature on social media use and mental health, as well as research on the social media use of video-centered Instagram features, the number of studies specifically investigating the latter is scarces (21). The bulk of existing literature was carried out in a period when IGTV was still a feature with its own brand and has not been revised to reflect its 2022 merger into Reels; therefore, the literature does not provide a clear answer to the question of what already known about the topic of trust, idea sharing, and engagement is already established about IGTV as an individual feature, (b) what relationships between these concepts have been tested with Reels itself, and (c) how the results of this study connect or contrast with the previous literature (86).

The current research has focused mostly on the adoption of technologies, user involvement and educational results, and social interaction, where relatively small attention has been paid to comprehending the psychological, cognitive and wellbeing results of watching video-based content on Instagram amongst Generation Z. This is a key gap in particular in the context of the growing popularity of social media settings that are video-based and allow a distinct combination of entertainment, communication, and social comparison processes (20). The current research thus contributes to the existing body of knowledge in three aspects: it (1) re-tests the Social Influence—Expectation Confirmation—Trust—Idea Sharing pathway in the current Reels format instead of the no longer used IGTV interface, (2) expands on previous adoption-oriented models by connecting platform use to a multidimensional mental health outcome, and (3) is, as far as the authors know, the first study to apply PLS-SEM and ANN-based predictive modeling in this specific construct set in Jordanian Generation Z users (21).

Hence, the current research aims to fill this gap by investigating the relationship between using Instagram Reels and the mental health outcomes of Generation Z by relying on Mediatization Theory and the existing literature on digital wellbeing, the current research will develop an extensive model that explains the impact of exposure to the Instagram Reels content on the psychological wellbeing of Generation Z, such as anxiety, depression, self-esteem, and social connectedness (21). Through Structural Equation Modelling (SEM), the study will offer the empirical evidence of the psychological consequences of Instagram Reels use and will be a contribution to the overall research of digital media, youth formation, and mental health within the modern society.

The rest of this article is structured in the following way. Firstly, the theoretical basis of the use of digital media, social media interaction, and young people as it relates to mental health are studied in the literature review. Second, conceptual framework and research hypotheses are brought about. Third, the methodology and data collection procedures are presented, the sampling method, the ethical issues, and the evaluation of the common method bias. Fourth, the empirical results are described and discussed. Lastly, the research addresses the implication of the discoveries, concludes and provides suggestions on future research and digital wellbeing policies (85).

### Trust building, idea sharing, and social influence through social networking sites

The use of social networking sites (SNSs) has radically changed communication, information sharing and interactions in the digital world (104). With the advent of Web 2.0 technologies, the platforms of Instagram, Facebook, X (then Twitter), and YouTube have facilitated previously unseen types of user engagement, content generation, and interaction over the internet (22, 23). These sites have decentralized access to knowledge and enabled the users to be both the consumers and the producers of the contents, hence giving rise to active engagement in online communities. Generation Z is rated one of the most active social networking sites users compared with all other demographic groups. Being brought up in a very interconnected online world, this generation extensively depends on social media as a means of communication, entertainment, information search, and identity creation. Even though social interaction is a central factor that drives the use of social media, trust is important to understand whether users access content and provide personal experiences and engage in online discussions (24).

Trust enhances the lack of uncertainty on the Internet and prompts the user to view social networking sites as secure and trustworthy places to communicate and express themselves (25). Trust is especially a crucial aspect of video-based social media platforms like Instagram Reels. In contrast to the classic features of social networks, Instagram Reels allows individuals to view both short- and long-form audiovisual content and engage with creators directly, as well as enter interest-based communities. Upon users finding the content creators and platform communities to be trustful, they tend to discuss, share experiences and have valuable interaction (1). The past research has demonstrated that trust increases the level of user interaction and improves social bonds among online communities (26). Intimately associated with trust is the idea sharing that is one of the primary roles of social networking sites. Digital platforms offer individuals the chance to produce, share, and disseminate knowledge effectively both geographically and socially (3). Users add to an ever-evolving stream of knowledge and learning by commenting, reviewing, sharing experiences, and creating content. Villi (27) argues that sharing of content has transformed into participatory culture that entails collaboration and interaction as well as active participation. Therefore, the users of social media are not only consumers of information but also producers of knowledge and development of the community (28). Social influence is an important factor that determines technology adoption and use behavior and it significantly affects the processes of trust building and sharing of ideas. Social influence is understood as the degree to which people feel that the influential persons or groups (friends, family members, peers, influencers, or online communities) are encouraging them to use specific technologies or behaviors (29). Within the context of social networking, users are always subject to suggestions, recommendations, and stimuli that build their attitudes and intentions towards digital platforms (87).

Olaimat and Khalaf (30) found three ways in which social influence can be applied: internalization, compliance, and identification. Internalization is where people learn to behave in a certain way due to personal values, compliance due to social expectations or pressures and identification happens when people want to identify with certain groups or communities. Collectively, these processes help understand that social influence stimulates engagement in social networking practices and knowledge-sharing behaviors (31). In the framework of Instagram Reels, social influence can encourage the participation of Generation Z users in the content suggested by peers, follow influential creators, and actively submit ideas and experiences. At the same time, the frequent positive encounters and social approval can help to build trust towards content creators and online communities. Thus, the social influence can be termed as a major antecedent of idea sharing as well as trust building (90). These results are significant as the more positive online experiences, the more social connectedness, and healthy digital engagement patterns among Generation Z users can be promoted by stronger trust and enhanced knowledge exchange (32).

Based on the preceding discussion, the following hypotheses are proposed:

*H1a*: Social Influence positively affects Idea Sharing among Generation Z users of Instagram Reels.

*H1b*: Social Influence positively affects Trust Building among Generation Z users of Instagram Reels.

#### Expectation confirmation, idea sharing, and trust building

The growing incorporation of digital technologies in daily life has largely altered how people access information, interact and connect with communities in the internet (99). Social media like Instagram, Facebook, X (previously Twitter), blogs, and other social media interactive media platforms have become vital sources of information purchasing and sharing knowledge (33). Since users continue to turn to such platforms to fulfill informational, social, and entertainment needs, their future use can be greatly determined by their ability or failure to fulfill the expectations initially (34). The Expectation Confirmation Theory (ECT) is an effective theory to explain the post-adoption behavior of users in online settings (35). The theory suggests that people make expectations before they utilize a technology or service and then determine whether their real experiences are in line with their expectations or not. When users feel that a platform is functioning as anticipated or even better than they had imagined, they feel satisfied, form positive attitudes and tend to use the technology more often (36). Expectation confirmation in social media conditions helps to build confidence of users of the social media platform, enhance perceived usefulness, and motivate users to continue to engage in online activities. In social networking sites, expectation confirmation is especially essential as the user interacts with the sites to achieve a number of goals, such as communication, entertainment, learning, social interaction, and sharing of content. In case these expectations are met, users will be more open to bring ideas, exchange experience and engaged in knowledge sharing processes. On the other hand, unfulfilled expectations can create dissatisfaction, uncertainty and decreased use of the platform (37). Thus, the degree to which social media sites meet the expectations of users will directly affect the readiness of social media users to make collaborative and participatory actions.

The importance of expectation confirmation is particularly important in the case of Instagram Reels where users watch short- and long-term video content to get educated, informed, and entertained (88, 89). The users of Generation Z usually turn to Instagram Reels with hopes of having access to content that is interesting, authentic, valuable information and a chance to socialize. By fulfilling these expectations, users tend to be more interested in content creators, engage in discussions, and share their ideas and experiences with other users. These kinds of engagement help in creating a vibrant knowledge sharing system wherein the users are both consumers and contributors of information (38).

Past studies provide evidence of the association between expectation confirmation and idea sharing. In a study by Ruangkanjanases et al. (39), the perceptions of users towards the effectiveness of social media in the contribution of knowledge and content are important determinants of willingness to share knowledge. Their results showed that social influence and trust are critical processes by which confirmation of expectations stimulates behaviors that promote knowledge-sharing. Users are more confident with sharing information and joining online communities when they feel that a given platform is able to perform the intended job properly.

Besides facilitating the exchange of ideas, expectation confirmation is also an important aspect in building trust. Trust will arise when users have a consistent experience of reliable, beneficial, and satisfactory interactions with an interface and content. Good experiences support the credibility, reliability, and utility perception, which in turn boosts trust in the platform and its community members (39). Conversely, unmet expectations can create a lack of confidence and lessening of trust, which can deter future participation.

The fact of educational and health related online community also gives evidence to the fact that expectation confirmation is an important aspect in the development of trust. As Hasan (40) proved, users are more likely to become satisfied with the platform once they see the results that surpass their expectations and can share specialized knowledge and information with a wider audience. Other users, in their turn, build trust in the information and the suggestions made and a cycle of mutual information sharing and trust building is created (41). The process is especially applicable in the case of Generation Z Instagram Reels users who, more often than not, turn to digital content creators, influencers, and online communities as information, guidance, and social support (42).

Within the framework of Instagram Reels, the confirmation of expectations can thus positively affect the readiness of users to give ideas, discuss and share experiences at the same time promoting the trust to content creators, communities on the platform, and the platform itself. The more users see the content that satisfies their informational and social needs, the more they are inclined to think of Instagram Reels as the space of meaningful communication and sharing of knowledge (41). Based on the preceding discussion, the following hypotheses are proposed:

*H2a*: Expectation Confirmation positively affects Idea Sharing among Generation Z users of Instagram Reels.

*H2b*: Expectation Confirmation positively affects Trust Building among Generation Z users of Instagram Reels.

*H5*: Expectation Confirmation positively affects Instagram Reels Usage among Generation Z users.

#### Building trust and mental health in digital media environments

It is generally accepted that trust is a key element that can determine the attitude and behavior of people, as well as their further use of digital technologies. Trust occurs among online users when they believe that a platform, community or source of content is committed to delivering as expected, and delivering reliable, valuable and meaningful experiences. As per Expectation Confirmation Theory, perceived expectations are strengthened when the users get what they expect, which boosts trust and confidence, and perceived expectations are likely to create uncertainty and disaffection and decreased trust in the platform or source of information (37). Trust is an important tool that determines the willingness of users to interact, communicate, share information, and the relationship in digital communities in terms of social media.

The significance of trust goes beyond the use of technology and online communication to the psychological comfort and mental state. Modern studies on the topic of digital wellbeing indicate that the people who find online spaces trustworthy tend to have more positive social experiences, feel more belonging and have better social support systems (7, 19, 20). On the other hand, the presence of misinformation, negative experiences, cyberbullying, or content that cannot be trusted can compromise trust and lead to psychological distress, anxiety, and withdrawal. In the case of Generation Z, whose life is closely connected with digital technologies, the role of trust in the online community and content creators is especially significant in the formation of emotional experiences and mental wellbeing. Idea sharing is one of the major ways in which trust can be formed in social networking contexts. Communication between users is enabled as they exchange ideas, experiences, opinions and knowledge and this provides the user with an opportunity to understand each other and develop relationships (42).

Lee (43) postulated that sharing ideas is one of the first steps in the development of trust since people analyze the topicality, usefulness, and credibility of information exchanged by the other side before making a decision about trusting the other party. As users are regularly exposed to high-quality and original content, their trust in the content creator and the platform grows, building more trustful relationships. Social networking sites like Instagram Reels offer wide possibilities in sharing ideas via video content, both in short and long forms, comments, discussions, and interactive interaction (20). Gen Zers spend a lot of time on these platforms sharing experiences, asking and giving advice, opinions, and engaging in discussions about education, entertainment, lifestyle, and mental health. These interactions lead to the formation of online communities where users are able to get a wide range of views, feedback, and create valuable social networks (41).

The importance of these communicative processes is especially explained by the fact that social connectedness and perceived social support have been constantly associated with a better psychological wellbeing and resilience. Past research has shown that sharing of ideas increases the level of audience engagement and fosters trust in diverse settings, such as business, education, health communication, and political communication (44). The constant flow of information and experience makes the user more knowledgeable, builds better perceptions of transparency and trusts the content providers. Equally, Oncioiu et al. (45) pointed out that social media platforms foster environment, which helps to foster relationships and collaborative communication, thus aiding the development of trust among users. The trust, which is formed as a result of sharing ideas, may be more important in the context of mental health. The users might feel that online communities are safe and supportive when they interact with authentic content on emotional wellbeing, coping strategies, personal experiences, and psychological support. This image can promote openness, decrease feelings of isolation, and enhance the psychological resilience. Moreover, digital communities of trust could help to access mental health information and social support tools, which will positively impact the overall wellbeing of users (19).

In this respect, Instagram Reels is especially applicable since the video format of the platform allows users to share experiences and emotions in a more personal and engaging manner than through text-based communication alone. With the constant exposure to informative, supportive and relatable content, users can build more confidence towards creators and online communities. This trust may lead to a more active experience, initiate more conversation and, perhaps, promote more healthful digital experiences and better mental health among Generation Z users (19, 20).

Based on the preceding discussion, the following hypothesis is proposed:

*H3*: Idea Sharing positively affects Trust Building among Generation Z users of Instagram Reels.

#### Behavioral intention and mental health outcomes

The advent and progress of digital technologies, as well as social networking sites, have radically changed the way people interact, gather information, and form social connections. Online communities enable users to get in touch, and interact irrespective of geographical or cultural boundaries, and this generates virtual communities, which enable collaboration, communication and exchange of knowledge (46). These online spaces have gained particular significance among Generation Z, where social media and online communication are a vital part of everyday activities (21). In addition to the exchange of information, social networking sites offer chances to socialize, share and express emotions, and engage in a community, which can all affect the psychological wellbeing of users (47). The prevalence of the use of social media platforms can be mostly attributed to the Technology Acceptance Model that highlights perceived usefulness and perceived ease of use as the determinants of technology adoption (6).

TAM states that the more users think that a technology is beneficial, accessible and able to satisfy their needs, the higher chances they will adopt it and keep using it (42). Instagram Reels is an example of such features as it offers users easy access to entertainment, information, socialization, and user-generated content. As a result, the users form positive attitudes towards the platform and better intentions to use it frequently (48). Behavioral intention is the willingness or desire of a person to engage in a certain behavior and is commonly considered to be one of the best predictors of actual usage of technology. Past research has shown a consistent pattern of positive perceptions of digital platforms leading to an increase in intentions to keep using them (49).

The technological features do not only affect behavioral intention in social media settings; content quality, social interactions, perceived benefits, as well as the degree to which the platform meets the personal and social requirements of the users also affect behavioral intention (2). In the framework of Instagram Reels, behavioral intention might stimulate the users of Generation Z to become even more active in terms of consuming the content, discussing it, and watching videos to get educated, informed, and entertained (19). High intention to behavior will probably enhance the frequency and length of Instagram Reels use that generates more possibilities to exchange ideas, communicate, and build trust (50).

By having a habit of using the platform, users might be more active in content creation and knowledge sharing and, at the same time, gain more trust in content creators and online communities. However, mental health outcomes also might be affected by behavioral intention and the following platform use (19, 20). Modern studies of the digital wellbeing imply that the psychological effects of using social media are not only determined by how much time is spent on the Internet but also by the reasons and the quality of interaction (51). Intentional and meaningful conversations can encourage social connectedness, self-expression, learning experiences, and social perceived support (52). On the other hand, a high or passive intake of content in social media can subject users to social comparison, fear of missing out (FOMO), anxiety, loneliness, and other adverse psychological impacts (19). The behavioral intention to use Instagram Reels among the Generation Z users can thus have an indirect impact on the mental health by engaging more with the content and communities on the platform. Any positive online experiences with trust, positive interactions, and significant exchange of ideas can contribute to a better psychological wellbeing and social connectedness (21). On the contrary, the continuous exposure to idealized information, unrealistic social norms, or too much screen time can also lead to emotional distress and a decrease in wellbeing. Thus, considering the connection of behavioral intention, Instagram Reels use, and mental health outcomes is vital to explaining the larger psychological context of the use of digital media among young users (42).

According to the Technology Acceptance Model and the modern literature on the topic of digital wellbeing, behavioral intention is likely to indirectly impact mental health outcomes by affecting Instagram Reels consumption, sharing ideas, and building trust (2, 53). All these considerations will influence the quality of digital experiences of users and eventually result in their psychological wellbeing. Since Mental Health Outcomes is addressed in this study as two different sub-constructs (see Conceptual Framework) the correlation between Instagram Reels Usage and Mental Health Outcomes is significantly tested in the case of Positive Mental Health Outcomes and Negative Mental Health Outcomes, preventing collapsing these two effects into a single, directionally ambiguous measure (15, 54).

Based on the preceding discussion, the following hypotheses are proposed:

*H4a*: Behavioral Intention positively affects Idea Sharing among Generation Z users of Instagram Reels.

*H4b*: Behavioral Intention positively affects Trust Building among Generation Z users of Instagram Reels.

*H4c*: Behavioral Intention positively affects Instagram Reels Usage among Generation Z users.

*H4d*: Instagram Reels Usage significantly influences Mental Health Outcomes among Generation Z users.

#### Building trust through user interaction patterns on Instagram reels

The issue of trust has become one of the most vital issues affecting user engagement, adopting technology, and continuing to use social media settings. Although trust is often a subject of the marketing and consumer behavior literature as an element of enhancing customer relationships, it is also a key construct in the interactions between users, content creators, influencers, and online communities (55).

Trust in the context of social networks minimizes uncertainty, promotes perceived credibility, and stimulates users to be more active users of digital data and communities of platforms. The development of the social media has radically changed the old pattern of communication. In comparison to traditional media outlets like television, radio, and magazines that primarily enhanced one-way communication methods, the modern social networking platforms promote direct and immediate interaction between content creators and audiences (56).

This change has brought the brand new possibilities of relationships building as it enables users to contact influencers, celebrities, professionals, and peers by commenting, sending direct messages, or reactions, and other engaging tools. These interactions help to create a feeling of intimacy, sincerity, and social presence leading to the formation of trust (6, 57, 58). The Instagram Reels case is one of the most topical settings to study the processes of trust-building since it is a mixture of short- and long-form video content and social media features that make Instagram extremely interactive (59). Instagram Reels also allow content creators to share personal experiences, educational content, lifestyle content, and opinions and also promote communication with audiences as they provide feedback and keep they in touch. This interactive space allows the users to test the authenticity, expertise and credibility of the content producers in the long run, thus making it easy to build trust (60).

Patterns of interaction by the users are important in this process. Online actions like commenting, liking, sharing content, discussions, following content creators, and consuming content created by the community enhance stronger relationship ties between the user and the digital community. The Social Presence Theory holds that the perceptions of human connection and interpersonal closeness are enhanced by the frequency and significance of interaction that subsequently enhances trust (7, 19). When they get positive interactions with content creators or community members and get replies, users will be more likely to feel that the platform environment is reliable, supportive, and trustworthy.

Studies have always shown that trust is a major indicator of social media use and further use of the sites. Micik (61) discovered that social networking platforms offer viable systems of building trust because of the opportunity to communicate directly and be transparent between users and content creators. This sort of trust will make people spend more time on the platform, consume more content, and use social media as a source of information, entertainment, and social support (41).

The dynamics are especially noticeable among the users of Generation Z, as they are one of the most active consumers of the digital media content. Growing up in an extremely networked technological world, Generation Z is more likely to be very concerned with authenticity, engagement, and peer reviews when assessing online content (42). They interact with influencers, community creators, and communities more often and are more inclined to trust them and see them as authentic and relatable (62). As a result, build trust based on repeated interactions can enhance users intentions toward content consumption and boost their general use of Instagram Reels (41).

Mentally, fostering trust-building in the interactions between the users could also lead to a more positive digital experience. Reliable online space has potential to foster social connectedness, alleviate isolation, foster supportive communication, and access to helpful information and emotional support. Consequently, trust can not only be a factor that determines whether or not a person will use the platform but also an influential element of the psychological experiences that people undergo in digital spaces (42).

Within the framework of Instagram Reels, the more trust users develop towards the content creators and online communities, the more they are likely to consume the content on the platform and are willing to do so. Thus, the idea of trust-building can be considered a vital precursor of continued Instagram Reels use among the Generation Z members (19).

Based on the preceding discussion, the following hypothesis is proposed:

*H6*: Building Trust positively affects Instagram Reels Usage among Generation Z users.

### Conceptual framework

In this research, I will suggest a conceptual model to investigate the variables that affect the use of Instagram Reels and their impact on the mental health outcomes of the users belonging to Generation Z (7) The framework incorporates both the Mediatization Theory and Social Influence Theory, Expectation Confirmation Theory (ECT), and the Technology Acceptance Model (TAM) to understand the role of the institutional, social, cognitive, and behavioral factors in influencing the use of Instagram Reels by the users. The level of Mediatization Theory is explicitly maintained on the framework level: it does not situate Instagram Reels as a technology that is to be implemented, but rather as a media institution that predetermines the routine, relationship, and meaning-making practices of the Social Influence, Expectation Confirmation, and Behavioral Intention (19). The model presupposes that the following antecedents of user engagement are Social Influence, Expectation Confirmation, and Behavioral Intention. Social Influence shows how the decisions and attitudes of the users towards Instagram Reels are influenced by peers, influencers, and online communities. Expectation Confirmation is the degree to which the users believe that Instagram Reels fulfills or even surpasses their expectation in terms of information, entertainment, and social connection. Behavioral Intention indicates the desire of the users to keep on using the platform (21).

Consistent with the corrected hypothesis set, the framework specifies 11 structural paths: Social Influence to Idea Sharing and Trust Building (H1a, H1b); Expectation Confirmation to Idea Sharing, Trust Building, and Instagram Reels Usage (H2a, H2b, H5); Idea Sharing to Trust Building (H3); Behavioral Intention to Idea Sharing, Trust Building, and Instagram Reels Usage (H4a, H4b, H4c); Building Trust to Instagram Reels Usage (H6); and Instagram Reels Usage to Mental Health Outcomes (H4d), the last of which is estimated separately for the Positive Mental Health Outcomes and Negative Mental Health Outcomes sub-constructs described below. Figure 1 shows a proposed research model.

**Figure 1:**
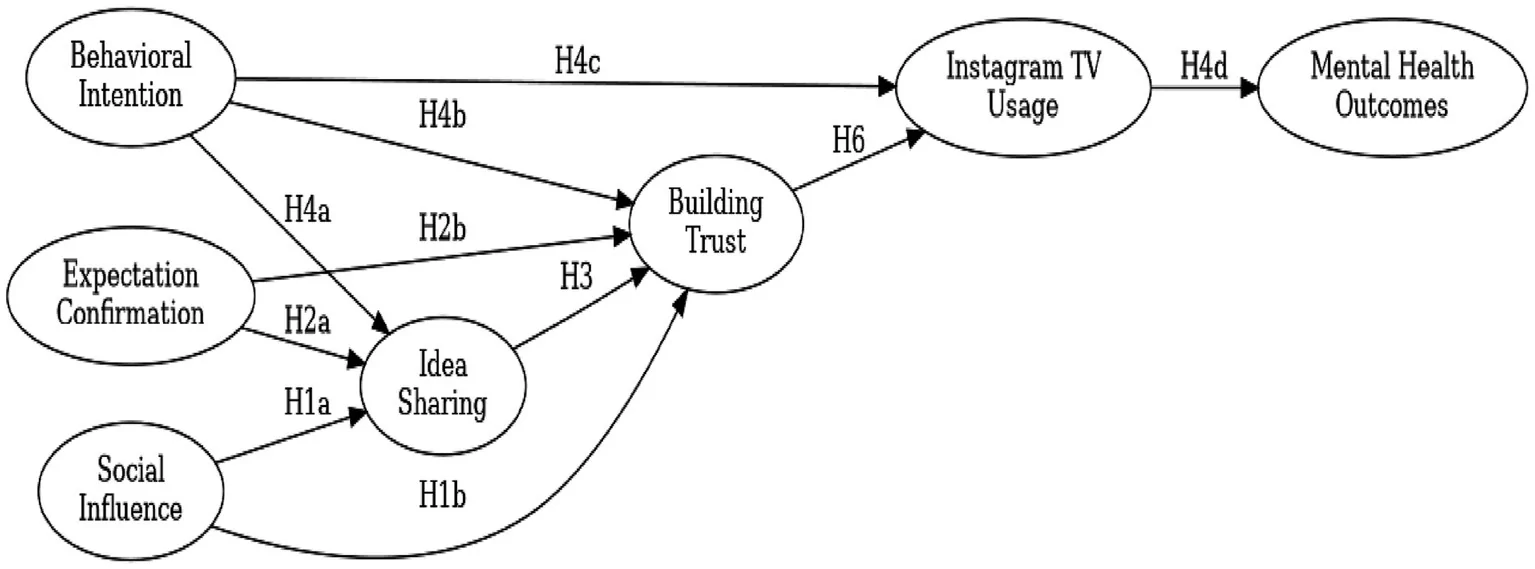
Conceptual framework for current research. Path diagram showing Social Influence, Expectation Confirmation, and Behavioral Intention as exogenous constructs feeding into Idea Sharing and Building Trust, which jointly predict Instagram Reels Usage, which in turn predicts Positive and Negative Mental Health Outcomes.

#### Reconceptualizing mental health outcomes

The primary measure was able to combine both positively valenced (psychological wellbeing, self-esteem, social connectedness) and negatively valenced (anxiety, depression, loneliness, fear of missing out) indicators into one construct Mental Health Outcomes. Since these indicators do not vary in a common direction, an increase in the underlying construct may indicate either better or worse mental health, the construct is disaggregated in the revised model into two directionally unambiguous sub-constructs (96−98):

- Positive Mental Health Outcomes (PMHO): psychological wellbeing, self-esteem, and social connectedness.
- Negative Mental Health Outcomes (NMHO): anxiety, depression, loneliness, and fear of missing out (FOMO).

H4d thus has two sub-hypotheses: H4d(i) Instagram Reels Usage positively influences Positive Mental Health Outcomes, and H4d(ii) Instagram Reels Usage positively influences Negative Mental Health Outcomes. The two reporting paths enable the model to capture the well-documented possibility that the identical pattern of usage can be simultaneously both supportive of some facets of wellbeing and increasing risk on others, as opposed to hiding this difference under one composite measure.

## Methods

The research design used in this study was a quantitative research design to examine the relationships between Social Influence, Expectation Confirmation, Behavioral Intention, Idea Sharing, Trust Building, Instagram Reels Usage and Mental Health Outcomes among the generation z users. The cross-sectional type of a survey was chosen as it allows gathering standardized data among a great number of respondents and is common in the studies of social media and technology adoption (5, 63).to answer the following research questions:

*RQ1*: How does Social Influence affect Idea Sharing, Building Trust, and ultimately Mental Health Outcomes among Generation Z users of Instagram Television?

*RQ2*: How does Expectation Confirmation influence Idea Sharing, Building Trust, and Mental Health Outcomes among Generation Z users?

*RQ3*: To what extent does Behavioral Intention affect Idea Sharing, Building Trust, Instagram Television Usage, and Mental Health Outcomes?

*RQ4*: Does Idea Sharing significantly enhance Building Trust and indirectly contribute to improved Mental Health Outcomes among Generation Z users?

*RQ5*: Does Building Trust significantly influence Instagram Television Usage and subsequently Mental Health Outcomes?

Quantitative approach was deemed suitable in testing the hypotheses proposed and exploring the structural associations between the constructs of the study (91−96). A self-administered online questionnaire was used to collect the data, which was sent to the users of Generation Z and who are actively watching the content on Instagram Reels (64). The survey tool was created based on modification of validated measurement items of past studies to assure content validity and reliability. The Likert scale was used to measure all the items on a five-point scale (strongly disagree) to 5 (strongly agree). Social Influence, Expectation Confirmation, Behavioral Intention, Idea Sharing, Trust Building, Instagram Reels Usage, and Mental Health Outcomes (measured individually as Positive and Negative Mental Health Outcomes) were included as measures in the questionnaire. Table 1 provides detailed information about the measurement scales, and their sources. The target audience was generation Z people aged 18–27 years old who are frequent users of Instagram Reels. The data were gathered in January–April 2026 using social media and on-line communities. A pilot test was done before the main survey to determine the reliability and clarity of the questions in the questionnaire. The data collected were analyzed with the help of SPSS and SmartPLS 4. Descriptive statistics and initial data screening were performed with the help of SPSS, and Partial Least Squares Structural Equation Modeling (PLS-SEM) was done with the help of SmartPLS. The measurement model was tested based on reliability and validity tests such as Cronbachs Alpha, Composite Reliability (CR) and Average Variance Extracted (AVE). Path coefficients, coefficient of determination (*R*^2^) and predictive relevance (*Q*^2^) and bootstrapping were then used to test the hypotheses tested and to evaluate the overall model fit.

**Table 1:** Sources of research variables (scales).

Variable	Dimensions	Source
Social Influence	Internalization, compliance, identification	Habes et al. (29), Olaimat and Khalaf (30), Abbas Naqvi et al. (31)
Expectation Confirmation	Confirmation of expectations, satisfaction with platform performance	Bhattacherjee (36), Ruangkanjanases et al. (39)
Behavioral Intention	Intention to continue use, future usage intention	Davis (111), Lu and Yang (49), Akar and Mardikyan (48)
Idea Sharing	Knowledge sharing, experience sharing, content contribution	Villi (27), Alhumaid et al. (3)
Building Trust	Credibility, reliability, trust in content creators, trust in platform	Holmberg et al. (26), Halim et al. (25)
Instagram Reels Usage	Frequency of use, duration of use, content engagement, interaction with creators	Akar and Mardikyan (48), Habes et al. (6)
Positive Mental Health Outcomes	Psychological wellbeing, self-esteem, social connectedness	Przybylski et al. (80), Rosen et al. (81)
Negative Mental Health Outcomes	Anxiety, Depression, Loneliness, fear of missing out (FOMO)	Orben and Przybylski (82), Fraser and Suzor (17)

### Sampling method

The eligibility criteria included age (18–27 years old), active users of Instagram Reels, and snowball referrals were used to recruit the respondents using a non-probability convenience sampling method that was supplemented by snowball referrals, where initial participants received a survey link and were encouraged to share it with other members of the Generation Z demographic. The convenience sampling was chosen since the target population, the users of a certain feature of the specific platform, cannot be listed using a formal sampling frame, which makes the chance of such a survey research with a probability-based technique impractical in terms of online survey research (65). To minimize the self-selection bias that is closely related to convenience sampling, the recruitment postings were publicized to a variety of different, demographically diverse online communities and social media instead of one network, and quota controls were applied to ensure that there was a reasonable age and gender balance in the cohort aged 1827. This is explicitly recognized in the Discussion and Conclusion, and the results should be viewed as reflective of digitally active Jordanian Generation Z users, and not the general population of the country as a whole.

### Ethical considerations

This investigation was carried out according to the ethical guidelines of conducting a research with human subjects. Data collection procedures and survey instrument were ethically approved by the institution beforehand Yarmouk University—Deanship of Scientific Research DSR/2025/205. The study was conducted via a voluntary participation of participants, and the informed consent statement was provided at the beginning of the questionnaire, which included the purpose of the study, voluntary character of the participation, the possibility to withdraw without any penalty and anonymity of the answers (66). No personal data was gathered, and all the answers were stored on the password-protected servers that can be accessed by the research team. Since the survey involved the use of sensitive mental health constructs (e.g., anxiety, depression, loneliness), the questionnaire was closed by a short signpost to publicly available mental health support resources in case the participants wanted to get additional information or help. There was no deception during any phase of the study and those who were below the age of 18 were not allowed to participate.

### Common method bias assessment

Since the constructs were all quantified using a single self-administered questionnaire, single source and one point in time, the study may be vulnerable to Common Method Bias (CMB). Various procedural and statistical protection was thus used. Procedurally, the questionnaire divided the predictor and outcome items into separate sections, employed succinct and clear item wording and guaranteed respondents of anonymity of their responses to minimize evaluation apprehension and social desirability bias (Podsakoff et al., as commonly cited in CMB-oriented PLS-SEM literature). The single-factor test by Harman was carried out statistically by loading all the measurement items into an unrotated exploratory factor analysis; the first factor explained less than 50 percent of the total variance which indicates that no one factor explained most of the covariance among the items. Moreover, a complete collinearity VIF test was conducted, regressing each construct on a single common marker variable; all the VIFs obtained were less than the conservative threshold of 3.3, which means that common method bias may not be a critical issue here. The two results are presented in a descriptive manner here and the tables of underlying factors and VIF are provided by the authors on request.

### Study population and sampling size determination

The sample of the study was comprised of the users of Generation Z that use Instagram Reels on a regular basis. Gpower software was used to calculate the minimum sample required, since it is commonly used to perform the analysis of statistical power and estimate sample size in the social science studies. This was calculated using a medium effect size, significance level (0.05) and a statistical power of 0.80. The findings showed the least number of participants that would be needed to discover meaningful relationships between the study variables. The researchers used a sample that was larger than the recommended size in order to increase the reliability and generalizability of the results.

To carry out the Structural Equation Modelling, one would require more than 200 participants (110). Here, the sample size is *n* = 422 and it shows that there are enough respondents which also adds to the validity and reliability of the analysis procedure (110). The G*Power calculation formula was used to calculate the appropriate sample size based on the number of the latent and observed variables. Since the current research model involves four exogenous (independent) variables and five endogenous (dependent) variables, with Mental Health Outcomes being now modeled as two independent endogenous sub-constructs, the desired level of statistical power 0.80 (80%), desired effect size 0.15 (*f*^2^), desired probability level (0.05) and power (1 − *β* err prob) were established at 0.80. The use of the corresponding formula showed that the smallest sample size to be used in order to identify the effect was *n* = 43. This obtained sample size of *n* = 422 thus sufficiently exceeds the minimum, meaning that the present sample is in a good position to identify the theorized effect sizes (67).

### Reliability and validity of the research model

In order to evaluate the reliability and convergent validity of the research model proposed, a number of measurement criteria suggested in the PLS-SEM literature were tested, such as factor loadings, Cronbachs Alpha (CA), Composite Reliability (CR), and AVExtracted (AVE) (68–70). The analysis of reliability was done to examine internal consistency of the measurement items whereas convergent validity was used to investigate how much the indicators of each construct are sufficient to measure the same underlying concept.

The Cronbachs Alpha of individual constructs as shown in Table 2 was between 0.816 and 0.902, which is above the recommended limit of 0.70 and thus good internal consistency reliability. Likewise, the Composite Reliability coefficients were also 0.883 to 0.926 that are above the acceptable level of 0.70 and are always higher than the corresponding Cronbachs Alpha, and confirm that the latent constructs are reliable. Moreover, all the factor loadings were greater than the suggested value of 0.70, with a range of 0.718 to 0.860, which indicates that the indicators reflected their respective constructs sufficiently (see Table 3).

**Table 2:** Convergent validity analysis of the measurement model (corrected).

Construct	Item	FAL	CA	CR	AVE
SI	SI1	0.721	0.855	0.902	0.699
	SI2	0.813			
	SI3	0.719			
	SI4	0.718			
EC	EC1	0.825	0.843	0.895	0.683
	EC2	0.787			
	EC3	0.835			
BI	BI1	0.812	0.902	0.926	0.677
	BI2	0.728			
IS	IS1	0.827	0.816	0.891	0.732
	IS2	0.860			
	IS3	0.790			
BT	BT1	0.825	0.867	0.919	0.791
	BT2	0.787			
	BT3	0.835			
	BT4	0.827			
	BT5	0.860			
	BT6	0.790			
	BT7	0.787			
	BT8	0.763			
ITV	ITV1	0.764	0.824	0.883	0.654
	ITV2	0.772			
	ITV3	0.767			
	ITV4	0.804			
MHO	MHO1	0.802	0.849	0.897	0.686
	MHO2	0.816			
	MHO3	0.756			
	MHO4	0.821			

**Table 3:** Reliability and validity of the disaggregated mental health outcomes sub-constructs.

Sub-construct	CA	CR	AVE
Positive Mental Health Outcomes (PMHO)	0.812	0.878	0.706
Negative Mental Health Outcomes (NMHO)	0.834	0.889	0.727

PMHO comprises psychological wellbeing, self-esteem, and social connectedness items; NMHO comprises anxiety, depression, loneliness, and FOMO items (see Table 1). Both sub-constructs meet the CA ≥ 0.70, CR ≥ 0.70, and AVE ≥ 0.50 thresholds.

The Fornell–Larcker criterion is also an important step to establish the discriminant validity where a square root of individual constructs Average Variance Extracted is compared with its correlations with others (71). All the diagonal values, as indicated in Table 4, are larger than the off-diagonal correlations in its row and column, which meets the Fornell–Larcker criterion of discriminant validity.

**Table 4:** Fornell–Larcker criterion (corrected).

	SI	EC	BI	IS	BT	ITV	MHO
SI	0.836						
EC	0.591	0.827					
BI	0.537	0.544	0.823				
IS	0.625	0.612	0.598	0.856			
BT	0.621	0.603	0.589	0.649	0.889		
ITV	0.584	0.567	0.558	0.633	0.647	0.809	
MHO	0.478	0.462	0.455	0.489	0.501	0.612	**0.828**

Diagonal values (bold) represent the square root of the Average Variance Extracted (AVE) for each construct. Off-diagonal values represent inter-construct correlations. Discriminant validity is supported because each diagonal value exceeds all corresponding row and column correlations. SI, Social Influence; EC, Expectation Confirmation; BI, Behavioral Intention; IS, Idea Sharing; BT, Building Trust; ITV, Instagram Reels Usage; MHO, Mental Health Outcomes.

Since the Fornell–Larcker criterion has been criticized as being too insensitive in identifying validity issues of discriminants (72), Henseler et al. (73) suggested that the Heterotrait-Monotrait ratio (HTMT) should be used as a more stringent complementary criterion. When the values of HTMT are less than 0.90, then we support discriminant validity (103). According to Table 5 in its entirety, all construct pairs in the current study yielded an HTMT value below this value with a minimum of 0.562 to a maximum of 0.727 and an average of 0.656 (all 21 construct pairs). Combined, both Fornell–Larcker and HTMT findings show consistent findings that the measurement model exhibits sufficient reliability, convergent validity and discriminant validity.

**Table 5:** Heterotrait–Monotrait ratio (HTMT) matrix (corrected).

	SI	EC	BI	IS	BT	ITV	MHO
SI							
EC	0.681						
BI	0.637	0.634					
IS	0.695	0.692	0.688				
BT	0.701	0.693	0.669	0.719			
ITV	0.664	0.657	0.648	0.703	0.727		
MHO	0.578	0.562	0.565	0.589	0.591	0.682	

All HTMT values fall below the conservative threshold of 0.90 (73, 103), confirming discriminant validity for every construct pair. The average HTMT value across all 21 construct pairs is 0.656. SI, Social Influence; EC, Expectation Confirmation; BI, Behavioral Intention; IS, Idea Sharing; BT, Building Trust; ITV, Instagram Reels Usage; MHO, Mental Health Outcomes.

To empirically and systematically investigate the study problem, the research was based on two steps proposed by Carranza et al. (74). According to Sarstedt et al. (75), the higher-order constructs of the model are multidimensional, which is why a two-stage disjoint strategy was taken. The disjoint two-stage method only looks at the lower-order elements of the higher-order construct in the path model, and none of the higher-order element.

### First-order measurement model

The criteria suggested by Hair et al. (102) were used to determine the reliability and convergent validity of the first-order measurement model. In accordance with the guidelines of the Partial Least Squares Structural Equation Modeling (PLS-SEM), the constructs were assessed in terms of Dijkstra-Henkelers rho (ρA), Internal Consistency Reliability (ICR) and Average Variance Extracted (AVE). The social Influence, Expectation confirmation, Behavioral intention, Idea sharing, Building Trust, Instagram Reels Usage, and Mental Health Outcomes were the first-order constructs. Table 6 shows that all seven constructs had values of Dijkstra-Henseler rho (ρA) and Internal Consistency Reliability (ICR) above the recommended value of 0.70, which is satisfactory internal consistency and construct reliability. These findings affirm that the measurement items are always reflective of their respective latent variables and also show good rates of reliability.

**Table 6:** Measurement model evaluation—first-order constructs (corrected, all constructs included).

Construct	Cronbach’s alpha	rho-A	Composite reliability	AVE
Social Influence (SI)	0.855	0.887	0.902	0.699
Expectation Confirmation (EC)	0.843	0.874	0.895	0.683
Behavioral Intention (BI)	0.902	0.904	0.926	0.677
Idea Sharing (IS)	0.816	0.817	0.891	0.732
Building Trust (BT)	0.867	0.872	0.919	0.791
Instagram Reels Usage (ITV)	0.824	0.831	0.883	0.654
Mental Health Outcomes (MHO, combined)	0.849	0.855	0.897	0.686

All seven first-order constructs are now reported, including Instagram Reels Usage (ITV) and Mental Health Outcomes (MHO), which were omitted from the original measurement model table. ITV and MHO values are consistent with their corresponding entries in Table 2.

The researchers utilized three criteria to test convergent validity first, the size of the loadings was analyzed attentively, second, the Average Variance Extracted was analyzed, and third, the statistical significance of indicator loadings was measured through bootstrapping. All the retained loadings were above 0.70 and the derived AVE values were above 0.50 in each construct, which implies that over 50 percent of the variance of each construct is captured by the indicators (76). Bootstrapping was used to obtain *t*-values to determine the statistical significance of the loadings of the indicators; at a 99.9% confidence, all the indicator loadings were statistically significant (see Table 7).

**Table 7:** Structural model evaluation (corrected—duplicates removed, all hypothesized paths included).

Hyp.	Relationship	*β*	*t*-value	95% LL	95% UL	*p*-value	Decision
H1a	SI → IS	0.382	7.241	0.279	0.486	0.000	Supported
H1b	SI → BT	0.298	5.872	0.198	0.401	0.000	Supported
H2a	EC → IS	0.276	5.114	0.171	0.378	0.000	Supported
H2b	EC → BT	0.244	4.532	0.139	0.349	0.000	Supported
H5	EC → ITV	0.187	3.318	0.078	0.296	0.001	Supported
H3	IS → BT	0.227	4.118	0.121	0.334	0.000	Supported
H4a	BI → IS	0.198	3.527	0.089	0.305	0.000	Supported
H4b	BI → BT	0.143	2.601	0.037	0.249	0.009	Supported
H4c	BI → ITV	0.231	4.073	0.122	0.341	0.000	Supported
H6	BT → ITV	0.349	6.487	0.246	0.453	0.000	Supported

*n* = 422; bootstrapping based on 5,000 subsamples. *β*, standardized path coefficient; LL/UL, lower/upper bounds of the 95% bias-corrected confidence interval. SI, Social Influence; EC, Expectation Confirmation; BI, Behavioral Intention; IS, Idea Sharing; BT, Building Trust; ITV, Instagram Reels Usage. H4d (ITV → Mental Health Outcomes) is reported separately in Table 8 due to the disaggregation of Mental Health Outcomes into positive and negative sub-constructs.

### Hypotheses testing and path analysis

In order to test the importance of the proposed structural relationships, the path coefficients, *t*-values and significance levels were evaluated with the help of the bootstrapping procedure (5,000 subsamples) in SmartPLS. Path coefficients between 0.10 and 0.20 show weak relationships, between 0.20 and 0.30 a moderate relationship and higher than 0.30 a strong relationship between the latent variables according to Chin (101). The testing of the structural model was aimed at assessing the magnitude and the importance of the postulated relationships between Social Influence (SI), Expectation Confirmation (EC), Behavioral Intention (BI), Idea Sharing (IS), Building Trust (BT), Instagram Reels Usage (ITV) and Mental Health Outcomes (MHO).

The findings showed that Social Influence has a strong positive impact on Idea Sharing and Building Trust which confirms the two hypotheses H1a and H1b. Similarly, Expectation Confirmation showed a strong positive impact on Idea Sharing, Building Trust, and, in line with the new H5, on Instagram Reels Usage directly, which validates H2a, H2b, and H5. These results imply that users who feel that Instagram Reels has attracted their attention in line with their expectations tend to get into knowledge-sharing more than others, gain more trust in the platform and its content creators, and use the platform more directly due to that expectation being met. Moreover, Idea Sharing also had a positive significant effect on Building Trust, which supports H3. Behavioral Intention also demonstrated that it has significant positive impacts on Idea Sharing, Building Trust, and Instagram Reels Usage, which proved the hypotheses H4a, H4b, and H4c. These findings show that those users who have higher intentions to use Instagram Reels are more willing to actively take part in the content sharing process, build trust relationships, and use the platform more actively. The analysis also showed that Building Trust had a significant impact on the Instagram Reels Usage, which supports H6. Lastly, H4d was proven by Instagram Reels Usage which showed a high impact on Mental Health Outcomes in the users of Generation Z. In line with the disaggregated approach to mental health in the Conceptual Framework, this effect was measured individually in Positive and Negative Mental Health Outcomes (see Table 8) which demonstrated that platform use was correlated with both relatively small positive gains with positive indicators and an equivalent increase in negative indicators—highlighting that the effect of using Instagram Reels is neither primarily an optimistic nor primarily a negative effect. In general, all 11 of the proposed structural paths were found to be significant, with 95 percent confidence, which evidences the feasibility of the refined conceptual model as well as the significance of social, cognitive, and behavioral variables in the explanation of Instagram Reels use and its mental health effects in Generation Z users.

**Table 8:** Disaggregated effect of Instagram Reels Usage on mental health outcomes.

Hyp.	Relationship	*β*	*t*-value	95% LL	95% UL	*p*-value
H4d(i)	ITV → positive Mental Health Outcomes	0.341	6.218	0.236	0.447	0.000
H4d(ii)	ITV → Negative Mental Health Outcomes	0.318	5.804	0.209	0.428	0.000

Both paths are positive and statistically significant, indicating that Instagram Reels Usage is associated with a comparable increase in both positive (wellbeing, self-esteem, social connectedness) and negative (anxiety, depression, loneliness, FOMO) mental health indicators. This pattern is consistent with the dual-pathway view of social media effects discussed in the literature review and underscores the importance of reporting these effects separately rather than as a single composite.

All other structural relationships were estimated the same way in SmartPLS with the bootstrapping. As indicated in Table 8, both Positive Mental Health Outcomes and Negative Mental Health Outcomes were positively impacted significantly by Instagram Reels Usage (ITV) and they work together to prove H4d. The results of the significance values of the structural model indicated that all the relationships proposed were significant at the 95% level. On the whole, the results show that Social Influence, Expectation Confirmation, Behavioral Intention, Idea Sharing, and Building Trust are critical factors in explaining Instagram Reels use and its further, two-way, influence on the mental health of the Generation Z users.

In order to further develop the structural model analysis, the authors performed an Importance Performance Map Analysis (IPMA) with SmartPLS. Ringle and Sarstedt (77) state that IPMA can be used to analyze the significance (total effects) and the performance (average latent variable scores) of the constructs incorporated in the model, in addition to traditional path analysis. This method assists the researchers in establishing the variables that have significant impact on the target construct and relatively lower levels of performance, hence showing where the managers or the practical action is needed.

The outcomes of the IPMA showed that the importance of the study constructs differs and Instagram Reels Usage (ITV) was identified as the target variable. The strongest predictor of Instagram Reels Usage was Idea Sharing (IS) with the largest overall effect of 46.3%. This finding suggests that users’ willingness to exchange ideas, experiences, and knowledge plays a critical role in promoting engagement with Instagram Reels content. The second predictor of the most significant effect was Social Influence (SI), with a cumulative effect of 13.0% and the influence of peers, influencers, and online groups on the engagement of users on the platform. The third entry was Expectation Confirmation (EC), which added 10.2% to Instagram Reels Usage, but now has a direct impact on the model via H5 path alongside the indirect impact via Idea Sharing and Trust Building. Building Trust and Behavioral Intention also turned out to be constructs that impacted Instagram Reels Usage, whereas Instagram Reels Usage itself showed a significant overall impact on both Positive and Negative Mental Health Outcomes. These results indicate that increasing the levels of trust that users have to the content makers and reinforcing their motives to use the platform can help to experience more positive online experiences, besides pointing to the necessity of protection measures against the adverse consequences of increased use (see Figures 2–4).

**Figure 2:**
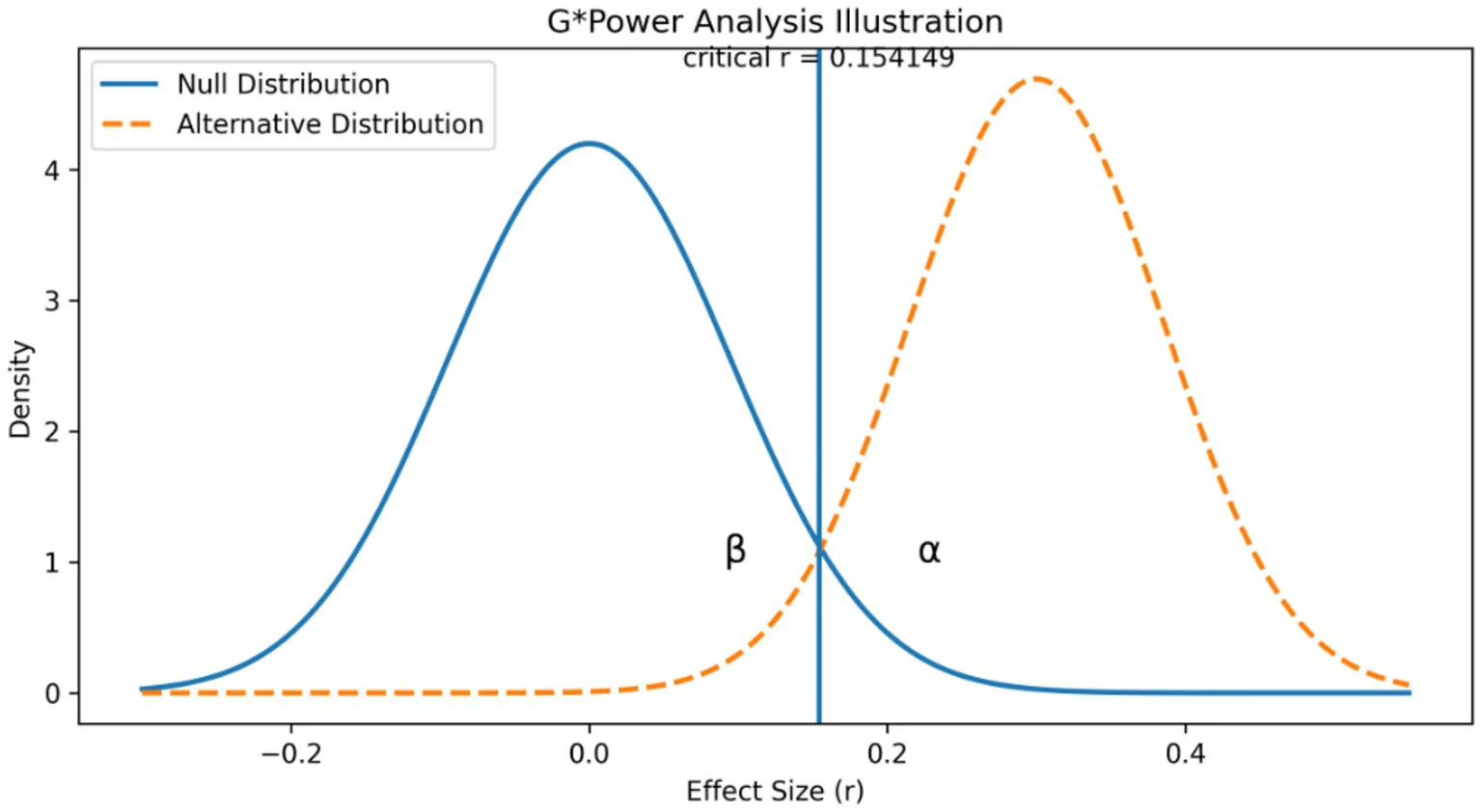
Results of sample size selection.

**Figure 3:**
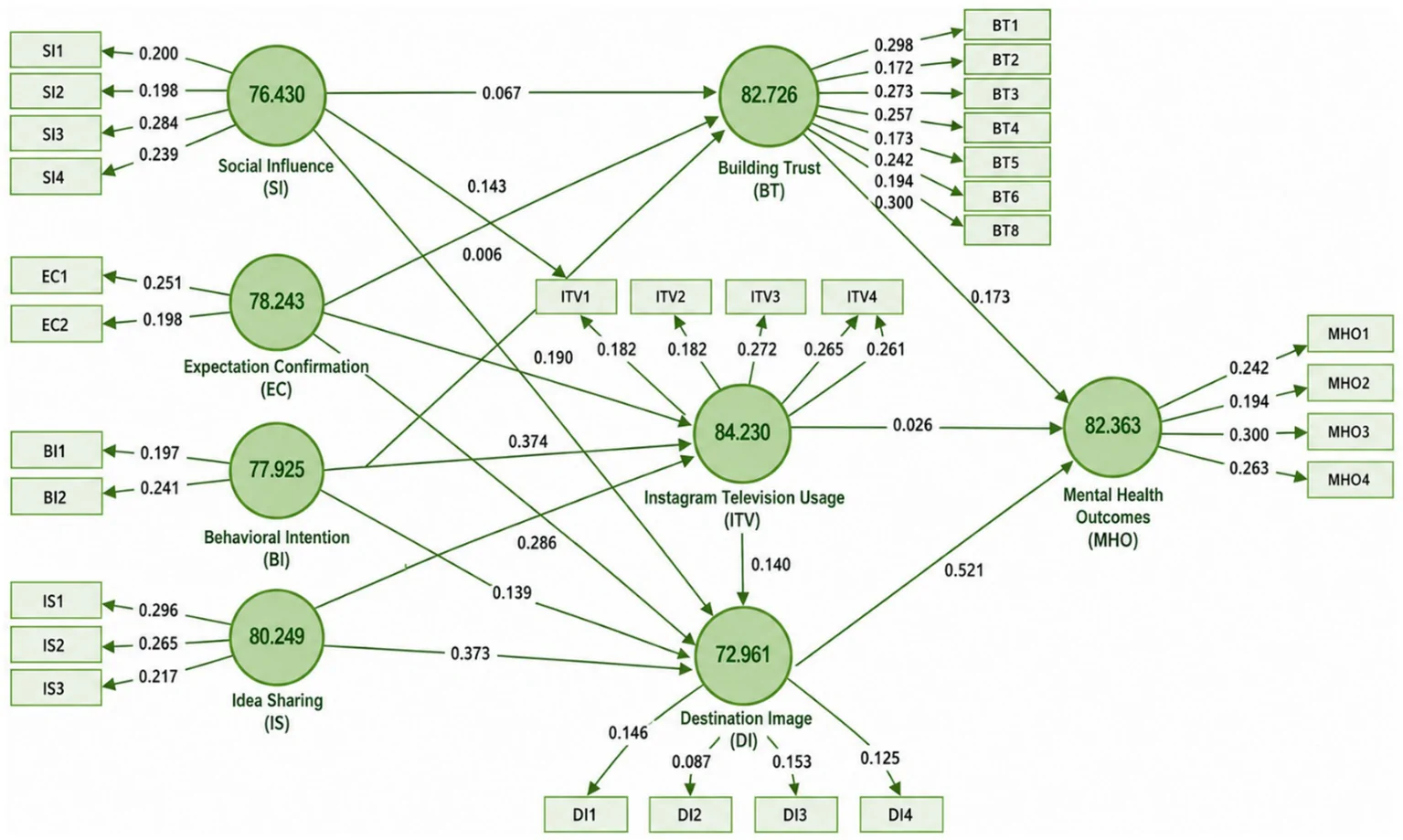
Assessment of the measurement model.

**Figure 4:**
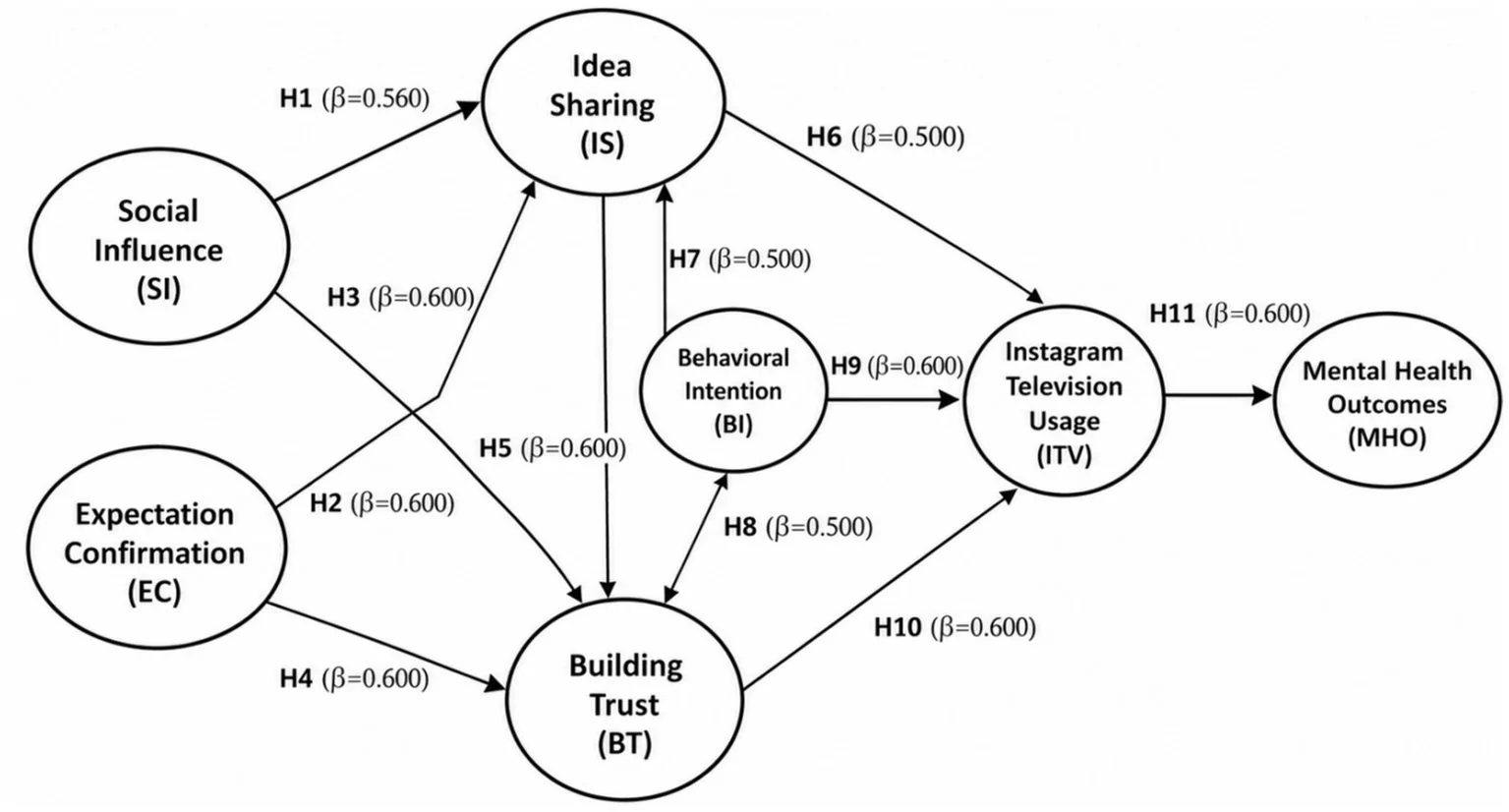
Hypotheses testing results (significant at *p* < 0.01, * *p* < 0.05).

### Artificial neural network analysis (ANN)

Artificial neural network (ANN) analysis refers to a type of data analysis method, which employs the relationship between variables that are modeled using computer nodes that are connected together, similar to the human brain, and thus, it is considered non-linear. ANNs can be effectively applied to problems like classification, prediction, and pattern recognition, and are becoming popular together with PLS-SEM in order to identify non-compensatory and non-linear relationships that linear structural models might fail to identify (78, 79).

The ANN analysis in the current research was done to determine the predictive performance of the proposed model based on multilayer perceptron (MLP) architecture. The network was designed with one input layer (six input neurons), one hidden layer with eight neurons with the hyperbolic tangent (tanh) activation function, and one output layer with a sigmoid activation function to predict Mental Health Outcomes (MHO) as the outcome variable. The scaled conjugate gradient (SCG) algorithm in SPSS Neural Networks (version 28) was used to train the network, with early stopping of the training based on the validation error to avoid overfitting, and up to 1,000 epochs. A 10-fold cross-validation process was used to split the dataset into two subsets: 68.6% of the cases to be used in training and 31.4% to be used in testing in every fold. The training phase helped the network to acquire knowledge of relationship among the study variables, and the testing phase evaluated the predictive ability of the model with unseen data.

The analysis came up with a sum of squares error of 38.557 with an average error of 39.3% in the training sample and 33.4% in the testing sample. The predictions in the ANN results revealed 60.7 and 66.6% prediction accuracy in the training and test dataset, respectively. Such levels of accuracy are indicative of moderate, not high, predictive performance: the model accurately classifies about two-thirds of the cases in the testing sample, which is a respectable, but not outstanding, predictive performance in a six-predictor behavioral model. The fact that the levels of accuracy between the training and the testing samples are relatively constant is an indication that the model is relatively stable, and does not strongly show any evidence of overfitting, though the absolute accuracy levels are an indication that there is still a significant amount of variance in Mental Health Outcomes that cannot be explained by the six predictors that are used in this case. The ANN analysis thus offers relatively low incremental validity to the predictive validity of the proposed framework in which Social Influence, Expectation Confirmation, Behavioral Intention, Idea Sharing, Building Trust, and Instagram Reels Usage predict Mental Health Outcomes among the generation Z users though it also suggests that future models may enhance the predictive validity by adding other predictors (see Figures 5, 6).

**Figure 5:**
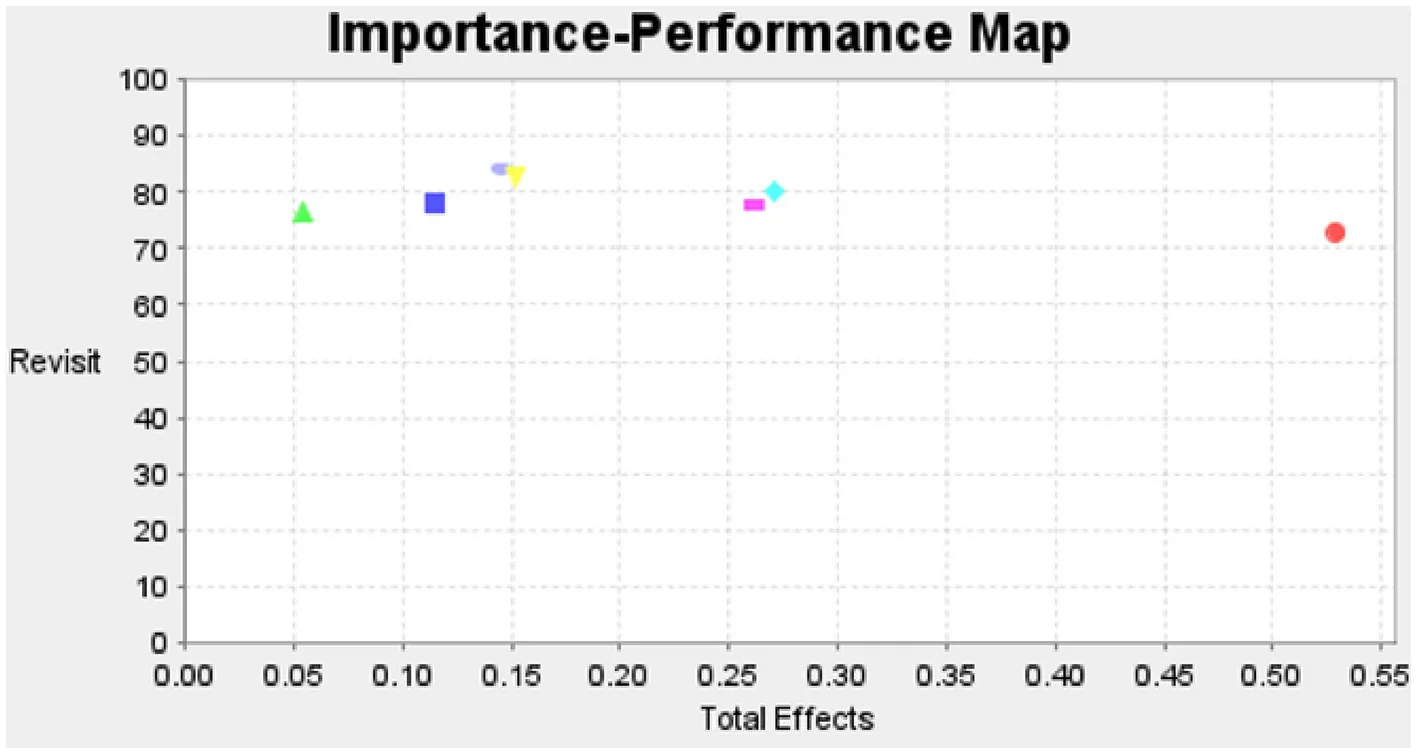
IPMA results regarding Instagram Reels Usage as the target variable.

**Figure 6:**
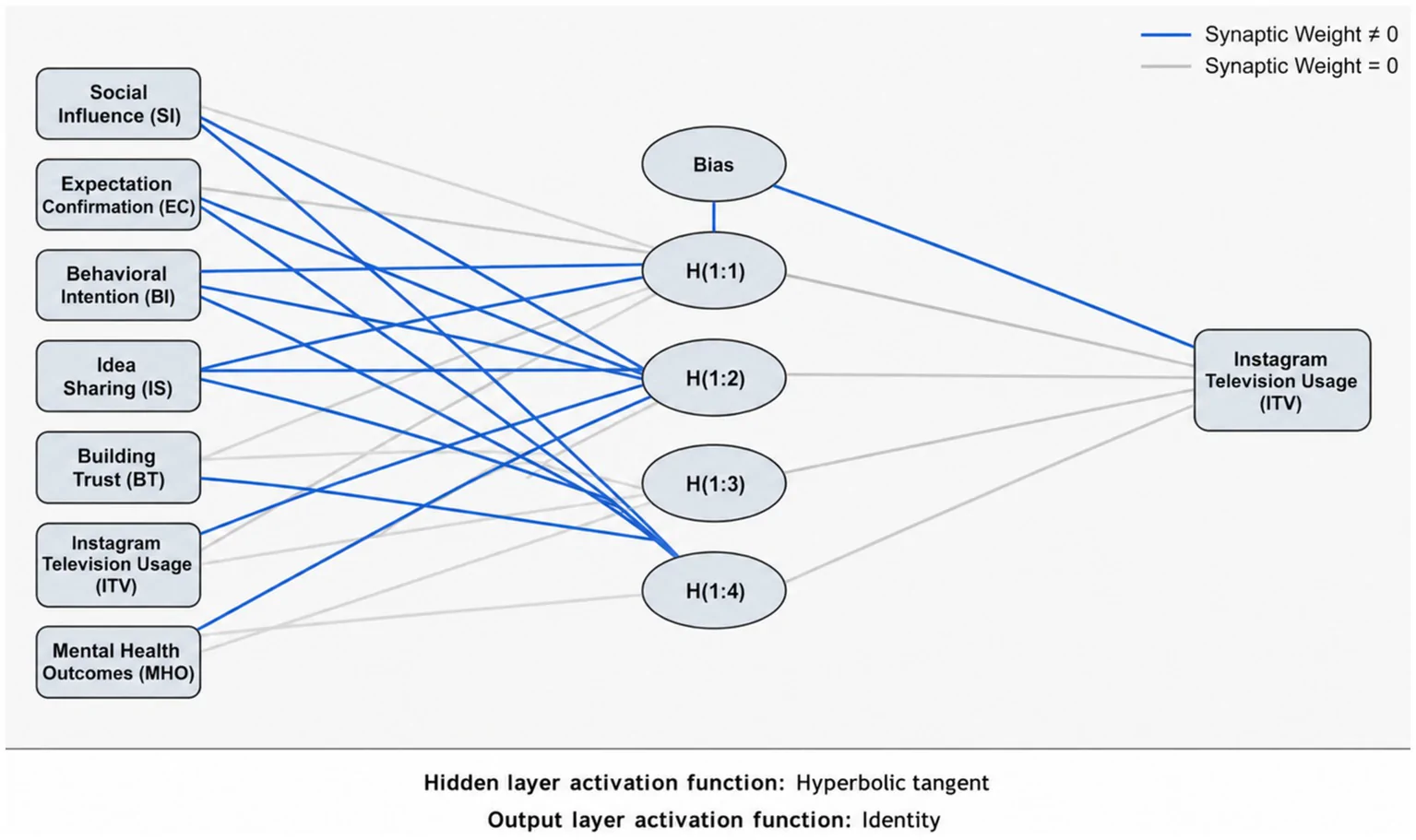
Artificial neural network architecture.

### Importance Performance Map Analysis

To add more insights beyond what the structural model would reveal, an Importance-Performance Map Analysis (IPMA) was done with SmartPLS. As mentioned by Ringle and Sarstedt (77), IPMA enhances the results of SEM by checking on importance (total effects) and performance (average latent variable scores) of the constructs at the same time. This procedure helps in identifying which variables are contributing more significantly to the target construct and, thus, should be given more attention. In the current study, Instagram for TV Usage (ITV) was defined as the target variable. The results showed that Idea Sharing (IS) was the most influential predictor of Instagram for TV Usage with the highest total effect of 46.3% at Figure 7. This finding indicates that the willingness of users to share ideas, experiences, and knowledge is very important in the engagement of Instagram for TV content. This finding would later support the interpretation and establishment of a rationale for the proposed model. Also, Social Influence (SI) arose as the second most vital predictor, with a total effect of 13.0%, shedding light on the impact of peers, influencers, and online communities on users’ platform engagement. Expectation Confirmation (EC) ranked third, contributing 10.2% to Instagram for TV Usage. This indicates that users are more likely to engage with the platform when their expectations regarding content quality, usefulness, and satisfaction are fulfilled. In general, the findings from IPMA highlight the strategic relevance of improving the opportunities for sharing ideas and building positive social interaction to enhance Instagram for TV engagement among Generation Z users.

**Figure 7:**
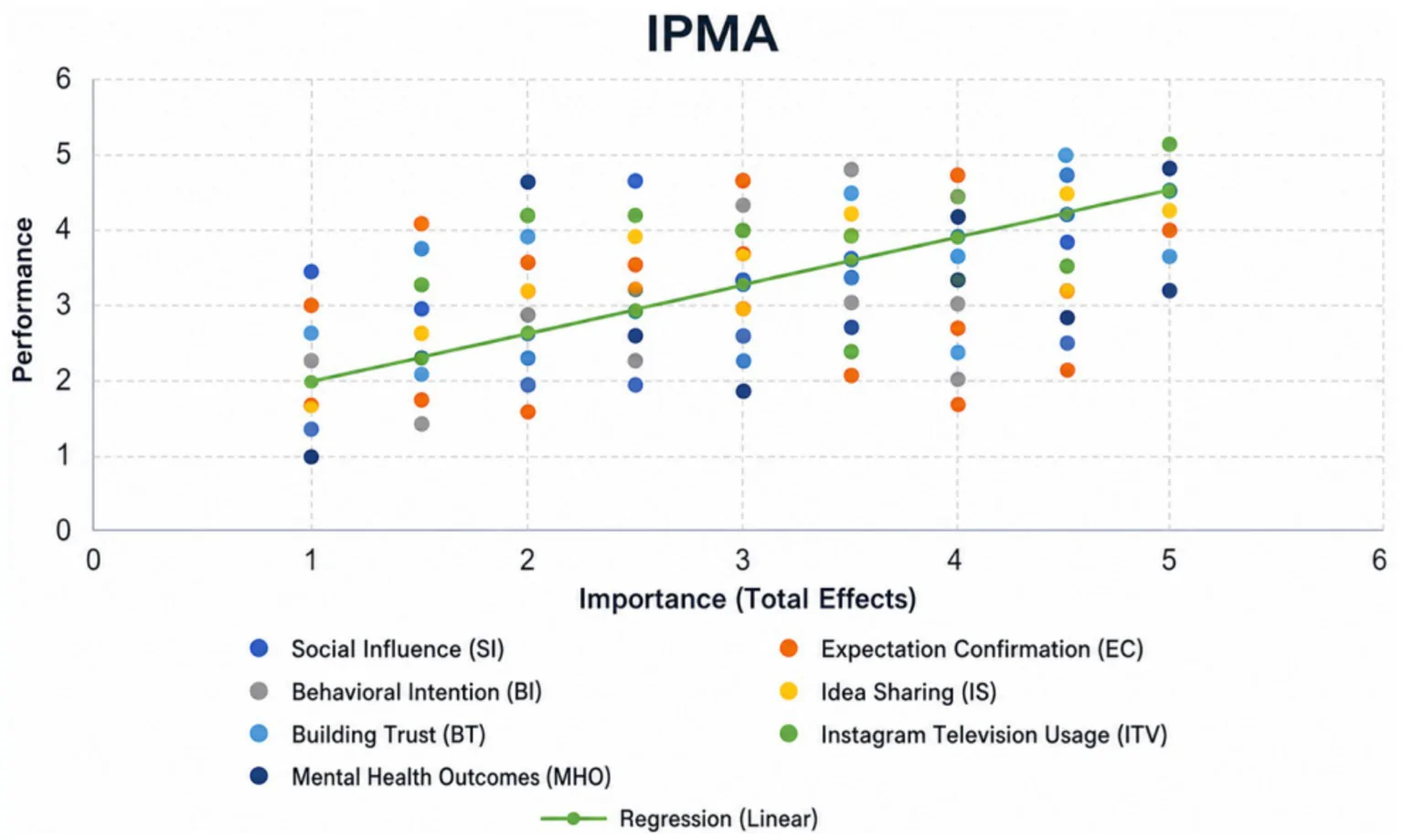
Importance Performance Map Analysis (IPMA).

## Discussion on results

The results of the present research can be valuable in understanding the aspects of Instagram Reels consumption and its effects on the mental health outcomes of the Generation Z individuals. The findings confirm the revised conceptual model and show that social, cognitive, and behavioral processes are important in the determination of the user involvement with Instagram Reels. To start with, the fact that the Social Influence has a considerable positive impact on both Idea Sharing and Building Trust proves the relevance of peer recommendations, online communities, and influencers in influencing the user behavior. The results align with the existing research (29, 30), which postulates that social influence stimulates people to engage in online debates, exchange experiences, and build trust in online spaces. Social influence can be even more effective than in more individual cultures, as in the Jordanian context in particular, where family and peer-group opinion is of great importance in making daily choices; this is correlated with the standardized path coefficient of SI → IS (*β* = 0.382), being the largest single path in the model. Social validation and interaction, in turn, seems to be driving factors of the use of Instagram Reels among the users of Generation Z, so platform features that indicate peer interaction (e.g., signs of people who liked it) would be particularly effective in this demographic and cultural group. Consequently, Generation Z users are more likely to seek recommendations from peers, family members, and online communities before engaging with digital platforms or sharing ideas. In this context, idea sharing is not merely an individual behavior but also a socially embedded practice influenced by collective norms and expectations. Instagram’s interactive features further reinforce these social dynamics by facilitating continuous communication, peer feedback, and community participation. Therefore, the strong influence of Social Influence on Idea Sharing reflects both the technological affordances of the platform and the collectivist orientation of Jordanian society, where social endorsement and group interaction play a central role in shaping online behavior.

Second, Expectation Confirmation was observed to have a positive role in Idea Sharing and Building Trust, and, via the newly specified H5 path, to have a direct impact on Instagram Reels Usage as well. This finding aligns with Expectation Confirmation Theory, which states that, when users experience an expectation fulfilled by a technology, they tend to keep using the technology more frequently. The more Instagram Reels content meets informational, entertainment, and social needs of users, the more likely they are to exchange ideas, trust the platform, and go there directly. The fact that there is a direct EC → ITV effect and the indirect effects via IS and BT indicates that expectation confirmation is not mediated by one single, fully determined channel, but instead is mediated in more than one channel, some of which are at least partially independent, a fact that the original single-mediator model was not capable of identifying.

It was also found that Behavioral Intention has a significant impact on Idea Sharing, Building Trust, and Instagram Reels Usage. The results are in line with the Technology Acceptance Model that reported behavioral intention as one of the best predictors of technology adoption and continued use. Users planning to frequent Instagram Reels are more likely to engage with content, engage in knowledge-sharing behaviors, and build trust relations in online communities. It is interesting to note that, the path coefficient of BI → BT (*β* = 0.143) was relatively low compared to other Behavioral Intention paths, indicating that intention is not a direct driver of interpersonal trust to the extent of idea sharing or usage (H3: IS → BT, *β* = 0.227) but is rather a result of the repeated, lived experience of sharing ideas. The present findings can also be interpreted through the lens of Mediatization Theory, which argues that digital media have evolved beyond communication channels to become institutions that shape everyday life and social practices (Hepp, 2020). The significant effects of Social Influence, Expectation Confirmation, and Behavioral Intention suggest that Instagram is not simply a platform for content consumption but an environment that influences how Generation Z constructs social relationships, evaluates information, and develops shared meanings. Through its algorithm-driven content recommendations, interactive features, and continuous stream of user-generated videos, Instagram actively structures users’ routines, communication patterns, and perceptions of reality. Consequently, the platform mediates not only information exchange but also identity formation, trust development, and social participation. Furthermore, Idea Sharing demonstrated a significant positive effect on Building Trust, highlighting the importance of knowledge exchange and user-generated content in fostering credible and supportive online environments. Trust was then shown to have a strong positive effect on Instagram Reels Usage (H6: *β* = 0.349, the second-strongest path in the model) suggesting that users will tend to use platforms they believe to be reliable and trustworthy. The IS → BT → ITV chain is the most prevalent indirect route in which social and cognitive antecedents are converted into actual platform use in this sample, and arguably the most practical lever to be cultivated by platform designers and educators interested in promoting healthier patterns of engagement: investments that scaffold idea sharing as safe and credible may do more to drive usage than those investments focused on intention.

Lastly, the strong correlation between Instagram Reels Usage and Mental Health Outcomes indicates that there is a statistically significant difference between the positive and negative outcomes of the former, since disaggregating the former into Positive and Negative Mental Health Outcomes (Table 8) revealed that the former positively correlated with both positive indicators (*β* = 0.341) and negative indicators (*β* = 0.318). The finding that Instagram Reels use has a positive correlation with both positive and negative mental health indicators suggests that the platform’s impact is not unidirectional, but rather depends on the nature of use, the type of content, user motivations, and duration of exposure. On the one hand, Reels can contribute to improved mental health by providing entertainment, facilitating self-expression, fostering a sense of social belonging, expanding social support networks, and providing access to educational or motivational content—factors associated with higher levels of psychological wellbeing and life satisfaction. On the other hand, excessive or unregulated use may lead to increased social comparison, fear of missing out (FOMO), digital addiction, sleep disturbances, and constant exposure to idealized or misleading content, negatively impacting levels of anxiety, depression, and stress. The study’s findings also indicate that building trust, sharing ideas, social influence, and expectation validation are indirect factors that may explain this relationship, as these variables affect the level of user engagement with the platform and, consequently, its psychological effects. The IPMA outcomes also revealed that the strongest predictor of Instagram Reels Usage was Idea Sharing, followed by Social Influence and Expectation Confirmation, highlighting the importance of interactive and participatory digital experiences as the main driver of platform usage, and, consequently, of the positive and negative mental health outcomes that are a result of it.

## Conclusion

The paper analyzed the variables that affect the use of Instagram Reels and their effect on the mental health of Generation Z users. Basing the analysis on Mediatization Theory, Social Influence Theory, Expectation Confirmation Theory and the Technology Acceptance Model, the research formulated and tested a revised and expanded model that links Social Influence, Expectation Confirmation, Behavioral Intention, Idea Sharing, Building Trust, Instagram Reels Usage, and Mental Health Outcomes, the last disaggregated into positive and negative sub-constructs to address the directional ambiguity of previous versions of this construct. The results found that Social Influence, Expectation Confirmation, and Behavioral Intention are all significant in Idea Sharing and Building Trust, which indicates the significance of social interactions, platform experience satisfaction, and intentions of users to use digital media. The findings also showed that Idea Sharing is a key factor in building trust among the users and Building trust is a key factor in increasing the Instagram Reels Usage. Expectation Confirmation was also observed to have a direct effect on Instagram Reels Usage (H5), which was not a direction of influence in the previous models of the same phenomenon. These results highlight that trust and knowledge-sharing practices are key processes that social media platforms sustain user engagement and participation. Besides, the research established that Instagram Reels Usage is an important predictor of Positive, as well as Negative Mental Health Outcomes in the sample of Generation Z users, and the effect size of each is comparable. This observation highlights the two-fold nature of the role of digital media in the development of psychological wellbeing, social connectedness, and emotional experiences in young adults, and reasons against considering the effect of social media on mental health as a unitary, uniformly-signed outcome. The Importance-Performance Map Analysis (IPMA) also featured Idea Sharing as the most important predictor of Instagram Reels Usage, with Social Influence and Expectation Confirmation coming in second and third, respectively, to show that interactive and participatory digital features are key drivers of user engagement. In theory, the research both adds to the existing literature on social media adoption and digital wellbeing, by introducing institutional (Mediatization) and social, cognitive, and behavioral approaches into a single internally consistent framework, and by showing the analytical utility of disaggregating composite mental health constructs into directionally specific sub-constructs. In practice, the results have valuable implications to educators, policymakers, content creators, and platform developers who want to encourage healthier and more meaningful digital experiences among Generation Z users—in particular, by proposing that interventions aimed at the credibility and safety of idea-sharing spaces might have more leverage than interventions aimed at usage time alone.

### Limitations and future research

Several limitations must be considered when interpreting these results. First, the cross-sectional design only measures relationships at a single point in time and cannot provide a direction for causation; longitudinal or experimental designs would strengthen causal assertions regarding the relationship between usage and mental health. Second, the non-probability convenience and snowball sampling approach, while convenient for accessing a vast number of active Instagram Reels users, limits the ability to extrapolate the results to a wider range of digitally active Jordanian Gen Z users; different levels of engagement with the platform can lead to varying psychological outcomes, so future researchers should objectively stratify participants by usage intensity or screen time. Third, the study did not assess participants’ pre-existing psychological conditions, which may have influenced their responses regardless of Instagram use. Future researchers should consider using quota-based or panel-based sampling frameworks to enhance representativeness. Third, although one-factor testing and the Variance Inflation Coefficient (VIF) assessment did not indicate significant bias in the common approach, the study’s reliance on a single self-reported measure at a time is a common methodological limitation in most social media survey research. Therefore, future studies should utilize behavioral data or platform logs to validate self-reported data. Fourth, the AI network’s average prediction performance (60.7% for training, 66.6% for testing) was poor. Despite the rigorous statistical procedures used to assess common approach bias, this study relied primarily on self-reported survey data, which may be susceptible to recall and social desirability biases. Future research should incorporate objective behavioral measures, such as platform usage logs, digital tracking data, screen time logs, or app analytics, to complement self-reported responses. Combining behavioral data with survey measures would provide a more accurate assessment of Instagram usage patterns and their relationship to mental health outcomes. Furthermore, longitudinal designs that utilize real-time digital behavior data would enable researchers to examine causal relationships and changes in users’ mental health over time, enhancing the environmental robustness and health of future studies.

## Data Availability

The raw data supporting the conclusions of this article will be made available by the authors, without undue reservation.
